# Exploring the Association Between Older Adults’ Body Mass Index and Their Fall Experience, Chronic Diseases, and Exercise Frequency: Evidence from Korea

**DOI:** 10.3390/medicina61091622

**Published:** 2025-09-08

**Authors:** Daekeun Kwon, Su-Yeon Roh, Jeonga Kwon

**Affiliations:** 1 Institute of Sports Health Science, Sunmoon University, Asan 31460, Republic of Korea; ksunsu@sunmoon.ac.kr; 2 Department of Exercise Rehabilitation, Gachon University, Incheon 21936, Republic of Korea; 3 Department of Elementary Education, College of First, Korea National University of Education, Cheongju 28173, Republic of Korea

**Keywords:** body mass index, chronic diseases, exercise, fall experience, older adults

## Abstract

*Background and Objectives*: Many older adults face health challenges, such as physical and functional decline, which increase the risk of hospitalization and dependence. As the global population ages, it is necessary to consider the health of older adults to avoid additional health burdens while improving their quality of life. This study aimed to explore body mass index (BMI) as a factor associated with health in older adults in South Korea. Accordingly, this study investigated the associations between the BMI of older Korean adults and exercise frequency (days per week), fall experience, and the presence of chronic diseases. *Materials and Methods*: Data from 10,014 older adults who participated in the 2023 Korean National Survey on Older Adults conducted by the Ministry of Health and Welfare were analyzed. Analyses included statistical frequency, chi-square tests, and multivariate logistic regression. *Results*: Underweight older adults had a higher likelihood of falling. Among those who were underweight, the odds ratio (OR) for falls was 2.052 (95% confidence interval [CI]: 1.349–3.121; *p* = 0.001). Underweight individuals were also less likely to engage in regular exercise. In contrast, both normal-weight and overweight individuals were more likely to participate in frequent exercise. Among those who were underweight, the ORs for exercising for 3–4 and ≥5 days were 0.612 (95% CI: 0.388–0.966; *p* = 0.035) and 0.721 (95% CI: 0.527–0.987; *p* = 0.041), respectively. Among those who were normal weight, the ORs for exercising for 1–2, 3–4, and ≥5 days were 1.286 (95% CI: 1.020–1.621; *p* = 0.033), 1.226 (95% CI: 1.055–1.424; *p* = 0.008), and 1.307 (95% CI: 1.167–1.464; *p* < 0.001), respectively. Among overweight individuals, the ORs for exercising for 1–2, 3–4, and ≥5 days were 1.275 (95% CI: 1.008–1.613; *p* = 0.043), 1.297 (95% CI: 1.114–1.509; *p* = 0.001), and 1.172 (95% CI: 1.042–1.318; *p* = 0.008), respectively. Older adults with obesity had a higher likelihood of having chronic diseases. Among those who were underweight, the ORs for one, two, three, and four diseases were 0.420 (95% CI: 0.268–0.658; *p* < 0.001), 0.335 (95% CI: 0.220–0.509; *p* < 0.001), 0.266 (95% CI: 0.167–0.422; *p* < 0.001), and 0.392 (95% CI: 0.254–0.606; *p* < 0.001), respectively. Among those with normal weight, the ORs for two, three, and four diseases were 0.686 (95% CI: 0.579–0.813; *p* < 0.001), 0.606 (95% CI: 0.505–0.727; *p* < 0.001), and 0.609 (95% CI: 0.505–0.735; *p* < 0.001), respectively, compared to those with obesity. Among those who were overweight, the ORs for two, three, and four diseases were 0.800 (95% CI: 0.671–0.953; *p* = 0.013), 0.781 (95% CI: 0.649–0.941; *p* = 0.009), and 0.686 (95% CI: 0.564–0.835; *p* < 0.001), respectively, compared to those with obesity. *Conclusions*: Weight is a key factor in promoting healthy aging in older adults. It is necessary to reduce the risk of falls in underweight individuals and prevent chronic diseases in those who are obese. Regular physical activity supports interventions that address these risks. Older adults should be supported and engage in consistent exercise tailored to their physical abilities and individual characteristics to maintain or improve their health and well-being later in life.

## 1. Introduction

Life expectancy has increased considerably in nearly all countries over the past century [1]. However, for many adults aged ≥60 years, living long does not necessarily mean living well [1]. Older adults face various health challenges, including physical and functional decline, hospitalization, and institutionalization-related disability, leading to a reduced quality of life, increased morbidity, and elevated mortality rates [2]. As the older population grows, their health and welfare impact society as a whole. Thus, the health of older adults and strategies that support healthy aging should be considered to reduce societal health burdens while improving their well-being. To design strategies for healthy aging, gaining a better understanding of the variables that affect the health of older adults is essential. Among these variables is the body mass index (BMI).

BMI is calculated by dividing weight by the square of height. Individuals are commonly classified into four categories: underweight, normal weight, overweight, and obese [3]. Specifically, using kilograms and meters: underweight or undernourished is <18.5 kg/m^2^, normal weight is 18.5–<25.0 kg/m^2^, overweight is 25.0–<30.0 kg/m^2^, and obese is ≥30.0 kg/m^2^ [3]. BMI is a simple, cost-effective, and noninvasive method for identifying weight status, as excessive weight (e.g., obesity) has been found to be associated with various morbidities and premature mortality across different ages, sexes, social groups, and ethnicities [4].

Such excessive weight, namely obesity, is characterized by the over-accumulation of fat in various parts of the body, including ectopic fat deposits. It is often caused by an imbalance between energy intake (food) and expenditure (exercise), leading to metabolic disruptions and insufficient energy use [5]. Globally, the prevalence of obesity continues to rise [6], driven by socioeconomic changes and lifestyle shifts. This is particularly true in South Korea. This trend is placing a growing burden on healthcare systems and is linked to increased risks, such as type 2 diabetes mellitus and cardiovascular diseases [6]. However, some studies point to the so-called “obesity paradox,” in which a higher BMI appears to have a protective effect among individuals with certain chronic conditions, such as chronic obstructive pulmonary disease, hypertension, and other comorbidities [7]. Conversely, being underweight has also been associated with negative outcomes in relation to chronic diseases [8]. For example, elderly underweight Korean individuals tend to have poor nutrient intake and a higher risk of anemia than normal, overweight, and obese groups; therefore, further diseases could be caused by anemia [9]. Chronic diseases, which are typically long-lasting and non-communicable, result from a combination of genetic, environmental, and lifestyle-related factors [10]. Therefore, we may gain new insights by exploring the relationship between BMI and chronic diseases among older Korean adults, given the multifactorial nature of these conditions.

Moreover, both underweight and obese BMI categories have been associated with an increased risk of falling compared to normal and overweight BMI [11]. Falls and fall-related injuries are prevalent among older adults, with approximately 30% of individuals aged ≥60 years experiencing at least one fall annually [12]. Xiong et al. [13] identified several key risk factors affecting the likelihood of falls in older adults, including demographic characteristics, physical function, chronic diseases, and psychological factors. The prevalence of falls varies across and within regions globally. For example, rates among ethnic Chinese populations in Southeast Asia range between 15% and 34%, whereas in Latin America and the Caribbean region, the prevalence ranges from 22% in Barbados to 34% in Chile [14]. Oceania has the highest fall prevalence at 34.4%, followed by the Americas at 27.9% [15]. Given that fall prevalence is influenced by race, geography, and culture, BMI among South Korean adults may also be associated with the likelihood of falling.

As a measure of weight status determined by balancing food intake and exercise, BMI underscores the importance of exercise. Regular physical activity offers substantial health benefits to individuals of all ages, and these benefits remain relevant later in life. Research suggests that exercise can extend the years of independent living, reduce disability, and improve well-being among older adults [16]. For this population, maintaining functional independence is as important as prolonging life, especially in terms of quality of life and effective healthcare resource management [17]. In other words, older adults can maintain autonomy in their daily lives through regular exercise. According to global guidelines, adults aged ≥65 years should engage in at least 150 min of moderate-intensity aerobic activity per week, along with two days of muscle-strengthening activities for meaningful health benefits [18]. However, despite the growing focus on health in response to global population aging, less than 15% of older adults meet the recommended levels of physical activity [19]. This highlights the need to encourage older adults to recognize the value of and adopt exercise practices. These practices can be tailored to individual physical conditions and consistently incorporated into their lives to support healthy aging. In fact, elderly people who exercise regularly can maintain high levels of activity for a long time, leading to a healthy lifestyle and improved quality of life [20,21]. Nevertheless, prior research offers limited and inconsistent evidence regarding the relationship between physical activity and BMI in older adults. Therefore, to fill this gap in the literature, our study examined the associations between BMI and the frequency of exercise, fall experience, and chronic disease among older adults in Korea. Our findings can serve as a foundational resource for the development of intervention strategies, as well as for health and welfare policies aimed at improving older adult well-being and promoting healthy aging.

Therefore, the purpose of this study was to investigate the relationship between BMI categories (underweight, normal weight, overweight, and obese) and fall incidence, physical activity frequency, and chronic disease prevalence in the Korean population aged ≥65 years. The hypotheses of this study were as follows: First, as the BMI of the Korean elderly increases, their experience of falls will increase. Second, as the BMI of the Korean elderly increases, chronic diseases will increase. Third, as the BMI of the Korean elderly increases, exercise frequency will increase.

## 2. Materials and Methods

### 2.1. Design and Study Population

To investigate the associations with BMI, we gathered data from the 2023 Korean National Survey on Older Adults conducted by the Korean Ministry of Health and Welfare. The survey assessed the living conditions and needs of older adults in various areas, such as health, family and social relationships, economic and social activities, financial status, and the housing environment. This study used stratified cluster sampling and covered a nationally representative population of 10,078 individuals aged ≥65 years. Both the survey’s sampling design and content received official statistical approval, and the study protocol was approved by the Institutional Review Board of the Korean Ministry of Health and Welfare (approval number: 117071; 31 December 2022). The Korean Ministry of Health and Welfare provides anonymized data from the survey after removing all personal identifiers (https://www.kihasa.re.kr/dataportal/kor/contents/ContentsList.html, accessed on 30 May 2025).

After we submitted the required documents, including a data request form, data use agreement (including a confidentiality statement), and a detailed research proposal, we were granted access to the SPSS dataset. In the population of 10,078 individuals aged ≥65 years, those with insincere or incomplete responses were excluded, and the resulting data from 10,014 participants were used. We used data from 10,014 participants and excluded those with insincere or incomplete responses. Design variables were used for complex survey sampling. All participants were informed of the purpose of the study and voluntarily signed an informed consent form. This study was conducted in accordance with the principles outlined in the Declaration of Helsinki.

### 2.2. Independent Variable

The independent variable in our study was the BMI of the participants. Following World Health Organization guidelines, participants were classified using an appropriate body mass index for Asian populations, as previously described [22]. BMI was calculated using the standard formula: BMI = weight (kg)/[height (m)]^2^. Based on Korean BMI classification standards [23], participants were categorized into four groups: underweight (<18.5 kg/m^2^), normal weight (18.5–<23.0 kg/m^2^), overweight (23.0–<25.0 kg/m^2^), and obese (≥25.0 kg/m^2^). Height and weight were recorded via self-report.

### 2.3. Dependent Variables

The dependent variables were the number of days of exercise participation, fall experience, and the presence of chronic diseases. We measured exercise participation by the question: “How many days per week do you exercise for at least 10 min?” The responses were open-ended and subsequently categorized into four groups for analysis: none, 1–2 days, 3–4 days, and ≥5 days. For the elderly, integrating physical activity into their daily lives is particularly important; therefore, the distinction of this category has been made from the perspective of physical activity habits. Being active ≥5 days/week was interpreted as consistent physical activity in daily life. Fall experience was assessed with the question: “Have you experienced a fall (e.g., slipping, tripping, or collapsing) in the past year?” The responses were binary and recorded as yes or no. The number of chronic diseases was measured by the question: “How many chronic diseases have you had for more than three months?” The answers were numerical and categorized as none, one, two, three, and four or more.

### 2.4. Statistical Analysis

SPSS for Windows (version 23.0; IBM Corp., Armonk, NY, USA) was used for data analysis. First, we conducted a frequency analysis of participant characteristics, such as sex, age, BMI, number of days of exercise, fall experience, and chronic disease. Next, we conducted a chi-squared analysis to identify differences in participant characteristics in terms of BMI. Finally, we performed multivariate logistic regression analysis to determine the association between BMI and the number of days of exercise, fall experience, and chronic disease. We calculated the odds ratios (OR), 95% confidence intervals (CI), and *p*-values. Statistical significance was set at *p* < 0.05.

## 3. Results

### 3.1. Participant Characteristics

In Table 1, among the 10,014 individuals, 5861 were male (58.5%) and 4153 were female (41.5%). In terms of age, 2932 participants were in their 60s (29.3%), 4549 were in their 70s (45.4%), and 2533 were over 80 years old (25.3%). Regarding BMI, 238 (2.4%), 3835 (38.3%), 3283 (32.8%), and 2658 (26.5%) participants reported being underweight, normal weight, overweight, and obese, respectively. Concerning the number of days of exercise, 4657 (46.5%), 532 (5.3%), 1505 (15.0%), and 3320 (33.2%) participants reported none, 1–2 days, 3–4 days, and ≥5 days, respectively. Regarding fall experience, 629 participants (6.3%) said that they had fallen in the past year, and 9385 (93.7%) reported that they had not fallen in the past year. Regarding the number of chronic diseases, 1360 (13.6%), 2175 (21.7%), 2752 (27.5%), 1975 (19.7%), and 1752 (17.5%) participants reported none, one, two, three, and four or more diseases, respectively.

### 3.2. Chi-Squared Tests

Table 2 presents the results of the chi-squared analyses. We found statistically significant differences in BMI in relation to sex (χ^2^ = 45.239, *p* < 0.001), age (χ^2^ = 149.317, *p* < 0.001), number of days of exercise (χ^2^ = 51.319, *p* < 0.001), fall experience (χ^2^ = 24.531, *p* < 0.001), and number of chronic diseases (χ^2^ = 69.550, *p* < 0.001).

### 3.3. Multivariate Logistic Regression Analyses

Table 3 presents the results of the multivariate logistic regression analysis of the relationship between BMI and fall experience. Regarding the relationship between BMI and fall experience, among those who were underweight, the odds ratio (OR) was 2.052 (95% confidence interval [CI]: 1.349–3.121; *p* = 0.001). In other words, the results revealed that underweight older adults were more likely to experience falls.

Table 4 presents the results of the multivariate logistic regression analysis of the relationship between BMI and the number of days of exercise. To determine the association between BMI and the number of days of exercise, the obesity group was used as the comparison group. Among those who were underweight, the ORs for 3–4 and ≥5 days of exercise were 0.612 (95% CI: 0.388–0.966; *p* = 0.035) and 0.721 (95% CI: 0.527–0.987; *p* = 0.041), respectively. Among those who were of normal weight, the ORs were 1.286 (95% CI: 1.020–1.621; *p* = 0.033), 1.226 (95% CI: 1.055–1.424; *p* = 0.008), and 1.307 (95% CI: 1.167–1.464; *p* < 0.001), respectively. Among those who were overweight, the ORs were 1.275 (95% CI: 1.008–1.613; *p* = 0.043), 1.297 (95% CI: 1.114–1.509; *p* = 0.001), and 1.172 (95% CI: 1.042–1.318; *p* = 0.008), respectively. These results indicate that underweight older adults were less likely to engage in physical activity than those with obesity, and older adults who were normal or overweight were more likely to exercise more frequently.

Table 5 presents the results of the multivariate logistic regression analysis of the relationship between BMI and the number of chronic diseases. Multivariate logistic regression analysis of the association between BMI and chronic disease used the obesity group as the comparison group. Among those who were underweight, the ORs for one, two, three, and four diseases were 0.420 (95% CI: 0.268–0.658; *p* < 0.001), 0.335 (95% CI: 0.220–0.509; *p* < 0.001), 0.266 (95% CI: 0.167–0.422; *p* < 0.001), and 0.392 (95% CI: 0.254–0.606; *p* < 0.001), respectively. Among those who were normal weight, the ORs for two, three, and four diseases were 0.686 (95% CI: 0.579–0.813; *p* < 0.001), 0.606 (95% CI: 0.505–0.727; *p* < 0.001), and 0.609 (95% CI: 0.505–0.735; *p* < 0.001), respectively; among those who were overweight, the ORs were 0.800 (95% CI: 0.671–0.953; *p* = 0.013), 0.781 (95% CI: 0.649–0.941; *p* = 0.009), and 0.686 (95% CI: 0.564–0.835; *p* < 0.001), respectively. Thus, the results showed that underweight, normal-weight, and overweight individuals were less likely to have chronic diseases than those who were obese.

## 4. Discussion

Our study aimed to determine how body weight, measured by BMI, is linked to the likelihood of falling, frequency of exercise, and number of chronic diseases among older adults in South Korea. First, we found that underweight older adults reported a greater likelihood of experiencing falls. Being underweight is also associated with negative health outcomes, such as increased disease burden, poor prognoses for various medical conditions, and heightened vulnerability among older populations [24]. Although a low BMI may offer some protective effects for middle-aged adults (aged 25–38 years), among individuals aged ≥65 years, it increases the risk of all-cause mortality by 57% [25].

Our data showed that underweight older adults had a significantly higher risk of falls than overweight or obese older adults. This finding contrasts with those of previous studies that emphasized obesity as a major risk factor for fall severity and frequency among older adults [26]. However, our findings are partially consistent with studies involving older adults, in which the underweight group also demonstrated a higher fall risk [27]. Although we could not identify the exact mechanisms linking underweight status to falls, the literature suggests that underweight individuals often exhibit poor body composition, impaired mobility, and decreased balance, all of which contribute to a higher fall risk [28]. Furthermore, the universal metabolic rate, which declines with age, was kept constant by decreasing BMI for several years as a signal of the final stage of lifespan [29]. Therefore, intervention strategies are needed in South Korea to identify and address the underlying causes of falls among underweight older adults to reduce fall-related injuries.

Second, we found that underweight older adults were less likely to engage in physical activity than older adults with obesity. Conversely, older adults who were either normal or overweight were more likely to exercise more frequently. This finding suggests that obesity is associated with reduced participation in physical activity. The findings of the few studies that have examined the relationship between physical activity and BMI in older adults are inconsistent [29,30]. The negative association between obesity and exercise participation that we found aligns with the findings of Sallinen et al. [29], who reported that older adults with obesity were significantly less active than their non-obese counterparts. The most frequently reported barriers to physical activity in obese individuals were a lack of motivation, pain or discomfort, and limited time. Among the few available studies, researchers have identified walking as the preferred form of physical activity among people with obesity [30].

Older adults with obesity often face greater challenges in engaging in physical activity due to a higher prevalence of comorbidities, pain, fatigue, fear of falling or injury, and physical discomfort or insecurity [29], which may explain their lower levels of participation. Underweight older adults are less likely to participate in physical activity than those with obesity. Ferreira et al. [31] reported that underweight older adults demonstrated the poorest physical performance, which may account for their lower levels of exercise participation. In summary, both underweight and older adults with obesity tend to engage in physical activity less frequently, albeit for different reasons: physical weakness in the underweight population and physiological or psychological barriers in the obese population. In particular, underweight elderly individuals have a high risk of fractures due to low muscle mass; therefore, exercise programs should be designed to increase muscle and body weight. However, for overweight and obese elderly individuals, exercise programs should be designed to maintain basal metabolic rate and increase energy expenditure, considering the burden on the waist and lower limbs due to their weight, by utilizing low-impact exercises, such as aquatic workouts. Third, we found that underweight, normal-weight, and overweight individuals were less likely to have chronic diseases than obese individuals. This supports the widely documented association between obesity and an increased risk of chronic diseases. A systematic review and meta-analysis by Larsson et al. [32] identified strong causal links between elevated BMI and numerous chronic diseases, including type 2 diabetes mellitus, cardiovascular diseases, asthma, chronic obstructive pulmonary disease, gastrointestinal disorders, musculoskeletal conditions, multiple sclerosis, and various cancers (digestive system, uterus, kidneys, and bladder).

Our findings are consistent with those of previous studies, indicating a positive relationship between higher BMI and an increased prevalence of chronic diseases among older adults [8,33]. Obesity-related inflammation further exacerbates the disease burden and interferes with the effective management of chronic conditions [34]. However, some studies have introduced the concept of the “obesity paradox,” suggesting that, in older adults, obesity may not be severely detrimental and may even offer protective effects. For instance, older adults with obesity may survive obesity-related health risks owing to their inherent physiological resilience or muscle mass preservation [35,36]. Given the age-related risks of malnutrition, muscle loss, and declining strength, older adults with obesity may have reduced mortality risk in some cases [37]. During the COVID-19 pandemic, the Korean government implemented a distancing policy to prevent infectious diseases [38]. Although the global fatality rate was low, > 90% of deaths occurred among the elderly and those with underlying diseases [37]. In particular, overweight and obese elderly individuals were able to maintain better physical function than non-obese Korean individuals [37], which was likely because they had better muscle retention than normal and lean individuals [38]. As such, there are various views on obesity, and prospective studies on elderly people with obesity should be conducted to confirm the existence of the obesity paradox. Therefore, weight loss in this group may not always be beneficial. Studies have linked a significant decline in BMI from midlife to late life to an increased risk of developing mild cognitive impairment [39]. Furthermore, weight loss can induce malnutrition and sarcopenia, potentially leading to life-threatening outcomes [40].

Therefore, alternative approaches to prevent obesity-related chronic diseases in older adults need to be identified. Rather than focusing solely on weight loss, strategies should emphasize muscle preservation and metabolic health through targeted physical activity and dietary management. In particular, exercise serves as a non-pharmacological treatment for improving health and preventing metabolic conditions in overweight and obese individuals. In a one-year diet and exercise intervention study among older adults (mean age = 70 years), Villareal et al. [41] reported a 9% weight reduction along with improvements in frailty, physical performance, and function. Notably, these improvements were only achieved through a combined intervention of exercise and diet, which helped preserve lean body mass and bone mineral density [41]. Therefore, older adults with obesity should consider engaging in safe and tailored physical activity to reduce the risk of chronic diseases while maintaining muscle and bone health.

### 4.1. Practical Implications of Our Study

Our findings indicate that underweight older adults are more likely to experience falls and less likely to participate in regular physical activity, whereas those who are normal weight or overweight are more likely to exercise. Additionally, older adults classified as obese have a higher likelihood of developing chronic diseases. Notably, 46.5% of the 10,014 participants in our study reported no engagement in ≥10 min of physical activity. These results suggest that maintaining a normal or overweight BMI, rather than being underweight or obese, is likely to be more beneficial for healthy aging. However, abrupt weight gain in underweight individuals or excessive weight loss in obese individuals can lead to adverse health outcomes. Thus, physical activity is a safer and more sustainable strategy for managing BMI and related health risks in older adults. During the aging process, physiological changes such as reduced appetite, nutritional deficiencies, and physical inactivity can lead to muscle wasting and loss of lean body mass [42]. Therefore, increasing the proportion of muscle mass relative to total body weight through physical activity is particularly beneficial. Regular physical activity has been shown to reduce the risk of chronic diseases, such as cardiovascular disease, type 2 diabetes, obesity, and certain cancers [43]. Progressive resistance training is especially effective for older adults, as it helps increase muscle strength and combat sarcopenia. Studies have also shown that it significantly improves lower-limb muscle strength and bone mineral density in the hip/femur region, with robust evidence supporting strength improvement [44]. The guidelines for resistance training for the elderly published by the NSCA recommend starting with an intensity of 20–30% of 1 RM and progressing to 80% of 1 RM, performing 3 sets of 8–12 repetitions 2–3 times a week [45]. Since the primary goal of resistance training for the elderly is to maintain the muscles they currently have, it is essential to be cautious to avoid injuries due to excessive intensity. It is important to recognize that even among individuals with the same BMI, health status can differ depending on various factors, such as muscle mass, lean body mass, and exercise habits. Falls, chronic diseases, and other age-related conditions can be mitigated by improving factors such as muscle power and strength [46,47]. Therefore, individually tailored progressive resistance training programs should be considered to increase muscle mass, prevent age-related decline, and promote healthy aging.

### 4.2. Limitations

Although our study contributes to the literature by exploring BMI in older adults, it has several limitations that warrant mention. First, we calculated BMI based on self-reported height and weight rather than through direct measurement using scientific equipment. This may have introduced self-reporting bias and inaccuracies. In addition, the analysis did not consider age-related adjustments in BMI classification. In particular, older adults tend to exhibit tendencies toward recall bias, social desirability bias, and reporting bias. Moreover, in geriatric populations, standard adult BMI cutoffs are increasingly recognized as inappropriate, owing to changes in body composition, such as loss of height, lean mass, and increased fat redistribution with age. Second, participants were classified using an appropriate body mass index for Asian populations [22]. However, BMI classification does not consider body composition factors, such as muscle mass, waist circumference, or waist-to-hip ratio, body composition, sarcopenia, central obesity, fat-free mass, or physical function. Therefore, the use of BMI alone to define obesity remains controversial [48].

Third, we did not distinguish the association between the types of chronic diseases. As the chronic diseases included in the survey were wide-ranging, such as cardiovascular, endocrine, musculoskeletal, respiratory, neuropsychiatric, sensory, digestive, urogenital, and cancer-related conditions, we were unable to establish associations between BMI and specific diseases. Given the heterogeneity of diseases, collapsing them into a single count may obscure meaningful differences. Fourth, both fall experience and the number of exercise days were based on self-reported recall, which may have introduced memory-related errors or inaccuracies. Fifth, exercise frequency, intensity, time, and type are important; however, we only considered exercise frequency as a variable for exercise participation. Sixth, the factors influencing each variable were not set as controlled variables. For example, factors that influence fall experience include medications, vision impairment, assistive device use, and recurrent falls; however, these factors were not adjusted. Additionally, factors such as age, sex, socioeconomic status, comorbidity burden, functional status, medication use, and mobility assistance were not considered despite being possible confounders that could affect the study results. Seventh, our study conducted a secondary data analysis, which allowed the identification of associations between variables, but did not establish causal relationships. In future research, studies measuring BMI should be conducted scientifically rather than using self-reported methods. BMI, fall experience, and the number of exercise days should be studied using objective methods of tracking records rather than self-reporting. Finally, future research should separate the types of diseases to specifically explore their causal relationships.

## 5. Conclusions

The objective of our study was to assess the association between BMI and the frequency of exercise participation, fall experience, and chronic diseases in older adults. We found that underweight older adults were most likely to experience falls and least likely to engage in physical activity, whereas those who were normal or overweight were more likely to participate in regular exercise. Notably, older adults with obesity were more likely to have chronic diseases. By confirming these connections, we extended the literature on BMI and provided further evidence of an association between excessive weight and chronic diseases, specifically in South Korea. To encourage healthy aging, regular physical activity should be supported among older adults in South Korea, as it can reduce the risk of falls and chronic diseases. In particular, increasing muscle mass through structured and consistent exercise, especially strength training, can help Korean older adults restore physical function and enjoy a healthier and more independent lifestyle. The results of this study will be useful to physicians and policymakers in developing effective policies and treatment methods to improve the health of the elderly.

## Figures and Tables

**Table 1:** Participants’ characteristics (n = 10,014).

Variables	Categories	n (%)
Sex	Male	5861 (58.5%)
	Female	4153 (41.5%)
Age	60s	2932 (29.3%)
	70s	4549 (45.4%)
	Over 80	2533 (25.3%)
Body mass index	Underweight (<18.5 kg/m^2^)	238 (2.4%)
	Normal (18.5–<23.0 kg/m^2^)	3835 (38.3%)
	Overweight (23.0–<25.0 kg/m^2^)	3283 (32.8%)
	Obesity (≥25.0 kg/m^2^)	2658 (26.5%)
Number of days of exercise participation per week	None	4657 (46.5%)
	1–2 days	532 (5.3%)
	3–4 days	1505 (15.0%)
	≥5 days	3320 (33.2%)
Fall experience	Yes	629 (6.3%)
	No	9385 (93.7%)
Number of chronic diseases	None	1360 (13.6%)
	One	2175 (21.7%)
	Two	2752 (27.5%)
	Three	1975 (19.7%)
	Four or more	1752 (17.5%)

**Table 2:** Differences in the characteristics of the study participants based on body mass index.

Variables	Categories	Underweight (<18.5 kg/m^2^)	Normal (18.5–<23.0 kg/m^2^)	Overweight (23.0–<25.0 kg/m^2^)	Obesity (≥25.0 kg/m^2^)	χ2(*p*)
Sex	Male	112 (47.1%)	2154 (56.2%)	2057 (62.7%)	1538 (57.9%)	45.239 (<0.001 ***)
	Female	126 (52.9%)	1681 (43.8%)	1226 (37.3%)	1120 (42.1%)	
Age	60s	46 (19.3%)	995 (25.9%)	1052 (32.0%)	839 (31.6%)	149.317 (<0.001 ***)
	70s	81 (34.1%)	1709 (44.6%)	1485 (45.3%)	1274 (47.9%)	
	Over 80	111 (46.6%)	1131 (29.5%)	746 (22.7%)	545 (20.5%)	
Number of days of exercise per week	None	147 (61.8%)	1708 (44.5%)	1475 (44.9%)	1327 (49.9%)	51.319 (<0.001 ***)
	1–2 days	7 (2.9%)	204 (5.3%)	187 (5.7%)	134 (5.0%)	
	3–4 days	23 (9.7%)	567 (14.8%)	538 (16.4%)	377 (14.2%)	
	≥5 days	61 (25.6%)	1356 (35.4%)	1083 (33.0%)	820 (30.9%)	
Fall experience	Yes	32 (13.4%)	247 (6.4%)	180 (5.5%)	170 (6.4%)	24.531 (<0.001***)
	No	206 (86.6%)	3588 (93.6%)	3103 (94.5%)	2488 (93.6%)	
Number of chronic diseases	None	48 (20.2%)	551 (14.4%)	453 (13.8%)	308 (11.6%)	69.550 (<0.001 ***)
	One	40 (16.8%)	884 (23.1%)	754 (23.0%)	497 (18.7%)	
	Two	54 (22.7%)	1024 (26.7%)	905 (27.6%)	769 (28.9%)	
	Three	37 (15.5%)	707 (18.4%)	657 (20.0%)	574 (21.6%)	
	Four or more	59 (24.8%)	669 (17.4%)	514 (15.6%)	510 (19.2%)	

*** *p* < 0.001: assessed through chi-squared tests.

**Table 3:** Association between body mass index and fall experience.

Outcome Variable	Category	Body Mass Index	ORs	95% CIs	*p*-Value
Fall experience	Yes	Underweight (<18.5 kg/m^2^)	2.052	1.349–3.121	0.001 **
		Normal (18.5–<23.0 kg/m^2^)	1.038	0.844–1.276	0.724
		Overweight (23.0–<25.0 kg/m^2^)	0.935	0.751–1.166	0.552
		Obesity (≥25.0 kg/m^2^)	Reference		

** *p* < 0.01: assessed using multivariate logistic regression analysis; OR: odds ratio; CI: confidence interval, reference is the obesity group.

**Table 4:** Association between body mass index and number of days of exercise per week.

Outcome Variable	Categories	Body Mass Index	ORs	95% CIs	*p*-Value
Number of days of exercise participation per week	1–2 days	Underweight (<18.5 kg/m^2^)	0.559	0.256–1.224	0.146
		Normal (18.5–<23.0 kg/m^2^)	1.286	1.020–1.621	0.033 *
		Overweight (23.0–<25.0 kg/m^2^)	1.275	1.008–1.613	0.043 *
		Obesity (≥25.0 kg/m^2^)	Reference		
	3–4 days	Underweight (<18.5 kg/m^2^)	0.612	0.388–0.966	0.035 *
		Normal (18.5–<23.0 kg/m^2^)	1.226	1.055–1.424	0.008 **
		Overweight (23.0–<25.0 kg/m^2^)	1.297	1.114–1.509	0.001 **
		Obesity (≥25.0 kg/m^2^)	Reference		
	≥5 days	Underweight (<18.5 kg/m^2^)	0.721	0.527–0.987	0.041 *
		Normal (18.5–<23.0 kg/m^2^)	1.307	1.167–1.464	<0.001 ***
		Overweight (23.0–<25.0 kg/m^2^)	1.172	1.042–1.318	0.008 **
		Obesity (≥25.0 kg/m^2^)	Reference		

* *p* < 0.05, ** *p* < 0.01, *** *p* < 0.001: assessed through multivariate logistic regression analysis; OR: odds ratio; CI: confidence interval, reference is the obesity group.

**Table 5:** Association between body mass index and presence of chronic diseases.

Outcome Variable	Categories	Body Mass Index	ORs	95% CIs	*p*-Value
Number of chronic diseases	One	Underweight (<18.5 kg/m^2^)	0.420	0.268–0.658	<0.001 ***
		Normal (18.5–<23.0 kg/m^2^)	0.935	0.781–1.118	0.460
		Overweight (23.0–<25.0 kg/m^2^)	1.026	0.853–1.235	0.782
		Obesity (≥25.0 kg/m^2^)	Reference		
	Two	Underweight (<18.5 kg/m^2^)	0.335	0.220–0.509	<0.001 ***
		Normal (18.5–<23.0 kg/m^2^)	0.686	0.579–0.813	<0.001 ***
		Overweight (23.0–<25.0 kg/m^2^)	0.800	0.671–0.953	0.013 *
		Obesity (≥25.0 kg/m^2^)	Reference		
	Three	Underweight (<18.5 kg/m^2^)	0.266	0.167–0.422	<0.001 ***
		Normal (18.5–<23.0 kg/m^2^)	0.606	0.505–0.727	<0.001 ***
		Overweight (23.0–<25.0 kg/m^2^)	0.781	0.649–0.941	0.009 **
		Obesity (≥25.0 kg/m^2^)	Reference		
	Four or more	Underweight (<18.5 kg/m^2^)	0.392	0.254–0.606	<0.001 ***
		Normal (18.5–<23.0 kg/m^2^)	0.609	0.505–0.735	<0.001 ***
		Overweight (23.0–<25.0 kg/m^2^)	0.686	0.564–0.835	<0.001 ***
		Obesity (≥25.0 kg/m^2^)	Reference		

* *p* < 0.05, ** *p* < 0.01, *** *p* < 0.001: assessed through multivariate logistic regression analysis; OR: odds ratio; CI: confidence interval, reference is the obesity group.

## Data Availability

Publicly available datasets were analyzed in this study. These data can be found here: https://www.kihasa.re.kr/dataportal/kor/contents/ContentsList.html (accessed on 30 May 2025).
